# Correspondence

**Published:** 1885-03

**Authors:** 


### CORRESPONDENCE.

*My Dear Editor.*—Although my last letter was from Paris, I believe I had not then been here long enough to give you much of interest; so, before leaving, I give you a few short notes of my impressions of this place, from a medical standpoint.

In Paris I find every one studies a specialty, but yet no one calls himself a specialist; for be it remembered, to be known as a specialist, here, is a mortal professional sin.

This at first seems paradoxical, it is true, but I will explain. The system of hospital service is such, that the attending physician serves from two to three years in a department, and then takes up another, and so on till he completes the round of all. Applications for admission to all the hospitals must come through the "Bureau Central," where they are assigned to the particular hospital, or department thereof appropriate to their several cases. This offers great advantages to the physician who desires to pursue a certain line of clinical investigation; for he has only to notify the Bureau Central of his intention, and all the cases in that particular line are sent to his wards. But, when his two or three years are up, he proceeds to some other department of clinical work, and thus I found Charcot, not as expected (after publishing a work on Renal Pathology) still investigating renal diseases, but now in charge of the Nervous Department of the Salpêtrière, devoting his time chiefly to the hysterical and the epileptic. I had the pleasure of accompanying him through his wards, which are very extensive and exceedingly well conducted.

The Salpêtrière Hospital it will be remembered, is exclusively for women, and contains the vast number of 4,000 beds, and at present contains 3,000 patients under treatment. It is about 160 years old, built in terraces, not unlike private residences, scattered over, I should say, thirty acres of ground, intermingled with beautiful gardens. I went over the whole of it, even the kitchen, and the butchers' department, and I cannot speak too highly of the order, cleanliness and efficiency in its conduct. And this is the same in all the hospitals in Paris which I visited. The new Hotel Dieu Hospital is a very superior institution, which it should be, as it cost 45,000,000 francs, ($9,000,000). It contains between 600 and 700 beds. The main building encloses an open court, with very fine architectural effect, and from the outside project several wings. Its location just on the banks of the Seine, next to Notre Dame Cathedral, shows its solid architectural proportions from the outside, to very good advantage.

The hospitals in Paris are made very attractive to patients, and large numbers of paying patients go there by preference, and thus the accommodations have kept increasing, till now, I am credibly informed that, combined, they contain one bed to every seventeen of the whole population of the city. This seems scarcely credible when we remember that in London the ratio is only one in about two hundred.

With the old Ecole de Medicine, I was disappointed; in size and equipment, it cannot approach the Edinburgh University. The class-rooms are small and dingy, the museum of anatomy small, but the library is fairly complete. The Pathological Museum of Dupuystren, in an outside building, is well arranged, and contains considerable material, but is in no way to be compared with the Hunterian Museum in London.

The new buildings, however, in course of erection for the accommodation of the faculty, when completed, will amply amend, in point of room, for any deficiency existing in the old school. They are very extensive, covering two blocks. The main building, fronting on the Boulevard St. Germain, is a magnificent and imposing piece of architecture; built of white stone, with massive carved figures above either side of the main door-way, which is the only ornamentation attempted, save the upper third of the front, consisting of a system of long porch-ways, after the Doric system, which gives a fine effect.

This building surrounds the old Ecole de Medicine, except the front of the latter, which faces to the rear, directly behind the Boulevard St. Germain. The next block of buildings is more plain and irregular, though built of the same white stone, and cover the block to the rear of the main building and face the old Ecole. The practical classes in anatomy already occupy these, and as I visited them I noted quite a number of female students dissecting in common with the male students.

A few days since an ambulance committee was formed for the purpose of insuring immediate attention for people meeting with accidents or sudden illness in the streets. This grew out of the fact that recently a case of injury was delayed some hours in reaching the proper hospital for treatment, which resulted in the death of the patient before he received proper medical attention. I understand the committee intend to adopt the New York ambulance system.

In France, all public medical service is rigidly exclusive, no foreign medical man is allowed to hold any position on a hospital staff, or to occupy a chair in any teaching institution.

Our own distinguished Brown-Sequard refused for a number of years to comply with the requirements, and become naturalized, even when offered a chair in the "Facultie," and it was only after France became a republic that his scruples were sufficiently overcome to permit him to qualify and accept the proffered honor.

In concluding this communication, I would express the opinion that it would be wise for the profession in America to be thoroughly alive to dangers of cholera next season, and to use all measures of precaution against its importation. It is my belief that it is almost certain to break out, at least in Paris, on the advent of warm weather, for these reasons. Cholera exists now in Paris, constantly; it is true only a few cases, some four to six or eight per week, I note by the official reports, as an average since I came; but during the first two and a half weeks of November it reached about 80, and was only apparently checked by cold weather.

The weather has been very cold here during my stay; in fact it has snowed nearly every alternate day, and if such weather will not completely subdue the disease (which it has not), the chances are largely in favor of an outbreak early in the summer. Of course on the extent of the outbreak, or the range of its extension, it would be superfluous for me to express an opinion. I only state what I find to be the facts, and local conditions that exist in Paris, and leave your readers to draw their own deductions. CHAS. W. PURDY.

Paris, Jan. 17th, 1885.

---

SPRAY IN OVARIOTOMY.

*To the editors of the* CHICAGO MEDICAL JOURNAL AND EXAMINER—*Gentlemen:* The following is an extract from a recent work by Dr. Emmet (Emmet's Principles and Practice of Gynecology, p. 715):

"In this country I do not know of any prominent operator who now employs the carbolic acid spray."

This statement implies that the writer is not persuaded of the value of spray in ovariotomy. My own experience has led me to an opposite opinion. Indeed, I should not like to do a laparotomy for any purpose without antiseptic spray. I have been led to this conclusion by the results of one hundred and eighty-three cases of removal of cystic ovaries, of which I have lost only twenty-one; but more especially by the result of the last one hundred of these cases, only ten of which were fatal, while thirty-eight were consecutively successful. I feel that to omit the antiseptic spray would be to deprive the patient of one of the ready and efficient elements of success. As I can hardly hope for much better results than these I have cited and being quite content to let well alone, I shall hesitate before disturbing my present plan of operation by giving up a detail to which I attach much importance.

Very respectfully your obdt. servant,

JOHN HOMANS.

161 Beacon St., Boston.

February 17, 1885.

